# Optical Sensor, Based on an Accelerometer, for Low-Frequency Mechanical Vibrations

**DOI:** 10.3390/mi13091462

**Published:** 2022-09-03

**Authors:** Rodolfo Sánchez-Fraga, Margarita Tecpoyotl-Torres, Israel Mejía, Jorge Omar Mañón, Luis Eduardo Riestra, Jesús Alcantar-Peña

**Affiliations:** 1 Gerencia de Microtecnologías del Centro de Ingeniería y Desarrollo Industrial, CIDESI, Querétaro 76125, Mexico; 2 Centro de Investigación en Ingeniería y Ciencias Aplicadas de la Universidad Autónoma del Estado de Morelos, CIICAp-UAEM, Morelos 62209, Mexico; 3 GE, Advanced Technology Organization, Querétaro 76146, Mexico

**Keywords:** ANSYS, modal analysis, harmonic analysis, stiffness constant, oscillation amplitude, micromachining, silicon etching, optical test bench

## Abstract

This article documents the design, manufacture, and testing of a silicon inertial optical sensor for low-frequency (lower than 2 kHz) applications. Three accelerometer designs optimized by parameterization using Finite Element Analysis were considered. The accelerometers were manufactured and the one with the highest performance at low frequency was chosen for testing, which was attached to a steel package. The feasibility of using probes, based on micro-machined sensing elements, to measure mechanical vibrations with high resolution was also studied. The detection is performed with an air interferometer, eliminating the need for electric signals that are susceptible to electromagnetic interference and large temperature variations. From the fabrication technology using only a silicon wafer with both sides etched, the frequency response of the sensor, temperature operation (higher than 85 °C) and with a resolution of 17.5 nm, it was concluded that is achievable and feasible to design and manufacture an optical vibration sensor for potential harsh environments with a low cost.

## 1. Introduction

Low-frequency vibrations are given, for example, in the case of seismic waves [1] or piezoelectric vibration of energy harvester systems [2] for medical imaging [3], human body dynamics [4], vibration, and acoustic noise due to the radial force acting on the stator of an automobile’s motor [5] or other machinery, as well as in underground train-induced vibrations [6], among others. The vibration detection may induce the presence of risks, for which a decision or action needs to be taken.

Traditional vibration sensors are very large, generally made with piezoelectric technology. MEMS sensors have been developed with great advantages, such as their reduced size and weight [7]. At low frequencies, capacitive accelerometers are most used due to their high sensitivity, simple transduction, readout circuitry, low noise level, less immunity to temperature linearity, lower area, low power consumption, stable direct current characteristics [8], and amenability for feedback [9]. Their manufacture with no exotic materials also gives them advantages over other kinds of accelerometers, but due to the small allowable displacement at the tip, they require a very stiff feedback loop, which reduces the useful bandwidth and dynamic range [7]. In general, the quick deployment and the reasonable cost of ownership are considered in [10] as good reasons for evaluating fully integrated MEMS devices for high-speed automation equipment that monitors vibration.

Most vibration-sensing relationships tend to focus on the magnitude of the oscillation, not on absolute position tracking, so linear sensors such as MEMS accelerometers are sufficient for capturing motion information. Acceleration forces may be static, such as the constant force of gravity pulling at our feet, or they could be dynamic—caused by moving or vibrating the accelerometer [11]. The bandwidth and g-range values determine their end applications [12].

The accelerometer consists of a mass attached by means of flexible supports to an inertial frame, a shock absorber, and a mechanism by which displacement of the moving mass is recorded. Mechanical vibrations coupled to the inertial frame result in a displacement of the surface of the mass, on an axis, with respect to its resting position. Acceleration forces may be static, such as the constant force of gravity (g force), or dynamic, caused by moving or vibrating the accelerometer [13].

The effective stiffness constant of the beams contributes to determining the frequency responses of the accelerometers, making them suitable for several applications. Therefore, several beam geometries have been designed, such as fully symmetrical double-sided H-shaped beams [14], symmetrical double-sided serpentines [15], slanted beams [16], straight- and crab-leg serpentines, folded [17], L-shaped beams [4], spiral beams [18], folded beams [19], π-shaped springs (fully differential capacitive MEMS [20]), and folded beams with turns (comb accelerometer) [21], among others.

The work documented in this article refers to the design of an optical sensor based on a silicon accelerometer, which was selected among three options with different arm shapes, according to its performance in the simulations, as well as in the respective manufacturing processes. Its development is described in the following sections: In Section 2, the design of the optical sensor and the accelerometer, the encapsulation of the vibration measurement probe and the test bench are presented. In Section 3, the respective manufacturing and implementation processes are shown, while in Section 4, the results of the experimental tests are provided, including the evaluation of the mechanical vibration measurement probe using optical detection mechanisms and the accelerometer performance tests. Finally, in Section 5, some concluding remarks are provided.

## 2. Design of the Optical Sensor and Test Bench

**Figure 1:**
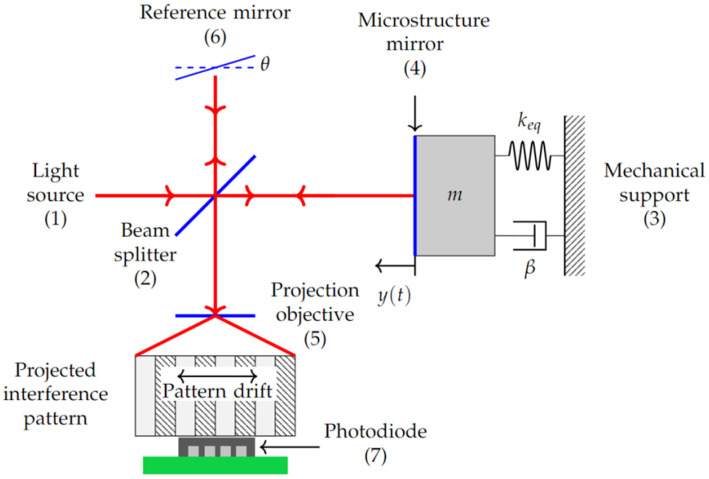
Conceptual model for optical vibration detection, with its elements numbered. Movement is considered in the Y axis.

The first step was to design three different accelerometers with the purpose of selecting the one that responded to a lower frequency, in addition to being feasible to manufacture at CIDESI. With the accelerometer or microstructure designed and manufactured, tests were performed on the arrangement shown in Figure 1. The surface of the accelerometer mass reflects a monochromatic, coherent beam of light, which allows the amplitude of its displacement to be measured using interferometric techniques (Figure 1).

For validation, a Michelson interferometer was used to analyze the interference between a reference laser beam and the beam reflected from the accelerometer surface (Figure 1). This interference is projected as a pattern of light and shadow bars onto a photodiode for evaluation using the measured electrical signal. To achieve this, the two mirrors have a tilt angle of π/2±θ to avoid a circular interference pattern and facilitate the photodiode conversion. When the microstructure surface mirror moves back and forth in the light direction, the pattern will exhibit a shift in the perpendicular direction, as shown in Figure 1, which is a function of the change in the distance between the beam splitter and the surface of the microstructure. When a bar takes the place of the adjoining one, it was known that the change in length was equal to half the wavelength of the light used.

Finally, for sensor testing, the interferometer was aligned with the shaker-mounted accelerometer for functionality testing. The shaker is used for exerting vibration effects.

### 2.1. Vibration Sensor Design

#### 2.1.1. Analytical Accelerometer Modeling

**Figure 2:**
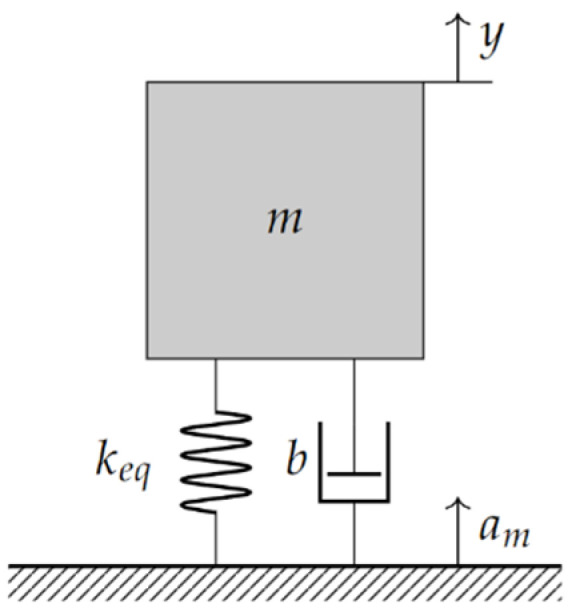
Basic mechanical Damped Mass-spring System Model.

In general, an accelerometer consists of a proof mass *m* supported by beam structures fixed to an inertial frame. The basic mechanical lumped model of the accelerometer is shown in Figure 2, where the supporting beams are modeled by an equivalent spring with a stiffness *k_eq_*, and dissipation effects are included using a damper with damping coefficient β.

A basic shape of springs is the rectangular guided beam, which could form part of more complex geometries. When a force *F* is applied, the change in position can be calculated by [22,23]:

(1) $$\Delta x=\frac{FL^{3}}{12EI}$$

where *L* is the beam length, *E* is Young’s modulus, and *I* is the second moment of inertia of the guided beam, which reflects the mass distribution of a rotating body or system of particles about an axis of rotation and is calculated by:

(2) $$I=\frac{ab^{3}}{12}$$

where a is the spring width and b is the thickness in the direction of the bending. From Hooke’s Law, considering Equation (1), the stiffness constant could be obtained [22,23]:

(3) $$k_{gb}=\frac{F}{\Delta x}=\frac{12EI}{L^{3}}$$

Arrangements in a series of parallel of rectangular guided beams can be used to form complex spring shapes. The effective stiffness constant of *n* springs in parallel, supporting the proof mass, can be calculated by Hooke’s Law, considering that the displacement is the same for all springs:

(4) $$k_{peq}=k_{p1}+k_{p2}+\ldots +k_{pn}$$

where $k_{p1},\;k_{p2},\ldots ,k_{pn}$ are the respective stiffness constants of the springs in parallel. In the case of four springs, each one contributes with ¼ of *k_eq_*, being commonly referenced in the literature as *k*_1/4_.

When there are *n* springs connected in series, the force actuating on each spring is the same. From the total displacement calculated by Hooke’s Law, the effective stiffness can be calculated as:

(5) $$k_{sn}={\left(\frac{1}{k_{s1}}+\frac{1}{k_{s2}}+\ldots +\frac{1}{k_{sn}}\right)}^{-1}$$

where $k_{s1}$, $k_{s2}$, …, $k_{sn}$ are the respective stiffness constants of the beams in series. Sometimes a single spring can be composed by several beams in series.

**Figure 3:**
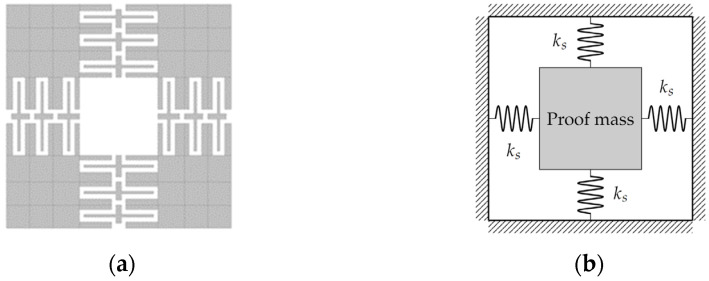
(**a**) Accelerometer with four arms composed by an arrangement of folded beams. (**b**) Equivalent diagram.

The calculation of the equivalent individual and total stiffness for the accelerometer, shown in Figure 3, is performed as follows.

The guided beam [22] or folded spring beam [24] shape is one of the most used in surface micromachining. The free end is allowed to move but not to rotate, leading to the beam bending into an S-Shape [22]. The folded beam can be considered as two guided beams in series, the small connecting parts can be neglected as its length is small compared to the large beams, and the spring constant is proportional to the length cubed. The stiffness constant of each beam can be calculated as:

(6) $$k_{b}=\frac{12EI}{L^{3}}$$

For two equal beams in series or folded beam, doubling the compliance, the stiffness constant is calculated as [22,24]:

(7) $$k_{1F}=\frac{k_{b}}{2}=\frac{6EI}{L^{3}}$$

Other approximation to the stiffness constant of single folded beam can be obtained from [25], or a simplified expression from [26]. The stiffness constant of two folded beams in parallel can be calculated as:

(8) $$k_{2F}=2k_{1F}=\frac{12EI}{L^{3}}$$

For the spring made by an arrangement of three folded beams in series, the stiffness constant can be calculated by:

(9) $$k_{s}=k_{3F}=\frac{k_{2F}}{3}=\frac{4EI}{L^{3}}$$

Comparing Equation (6) with Equation (9), it can be observed that the stiffness of the spring formed by three folded beams arrangements is 3 times higher than that corresponding to a single large beam.

Since there are four springs formed as folded beam arrangements attached to the mass, the equivalent constant for each beam is given by:

(10) $$k_{eq}=4k_{s}=\frac{16EI}{L^{3}}$$

On the other hand, the characteristic differential equation of the damped mass–spring system is given by:

(11) $$m\ddot{y}+\beta \dot{y}+k_{eq}y=F_{e}\;↔\;\ddot{y}+\frac{\beta }{m}\dot{y}k_{eq}+\frac{k_{eq}}{m}y=a_{m}\;$$

where *F*_*e*_ and *a*_*m*_ are the force and the acceleration, respectively, to which the system is subjected.

The transfer function of the second order, given by Equation (12), is the one-degree-of-freedom damped resonator equation and is obtained using Laplace transformations. This expression is equivalent to the one given in relationship with the quality factor [22].

(12) $$\frac{Y\left(s\right)}{a_{m}}=\frac{1}{s^{2}+2\xi \omega_{n}s+\omega_{n}^{2}}$$

with $\omega_{n}^{2}=\frac{k_{eq}}{m}$ or $f_{n}=\frac{1}{2\pi }\sqrt{\frac{k_{eq}}{m}}$, and $\xi =\frac{\beta }{2\sqrt{k_{eq}m}},\;$*ω*_*n*_ is the oscillation frequency of the first resonance mode, *ξ* is the damping ratio, and *m* = *ρV*, where *ρ* is the material density and *V* is the corresponding mass volume.

As a guide for the design, the undamped harmonic response was considered, (*β* = 0). From Equation (2):

(13) $$m\ddot{y}+k_{eq}y=F_{0}cos\;\omega t$$

The complete solution of this second order differential equation is given by the sum of the homogeneous and particular solutions:

(14) $$y\left(t\right)=y_{h}\left(t\right)+y_{p}\left(t\right)$$

with $y_{h}\left(t\right)=C_{1}\cos\omega_{n}t+C_{2}\sin\omega_{n}t$, and $y_{p}\left(t\right)=Ycos\;\omega t$

The maximum oscillation amplitude *Y* is given by:

(15) $$Y=\frac{F_{0}}{k_{eq}-m\omega^{2}}=\frac{ma_{m}}{k_{eq}-m\omega^{2}}=\frac{\frac{a_{m}}{\omega_{n}^{2}}}{1-\frac{\omega^{2}}{\omega_{n}^{2}}}$$

Therefore, the ratio between *Y* and the frequency is given by:

(16) $$Y=\frac{a_{m}}{\omega_{n}^{2}-\omega^{2}}$$

**Figure 4:**
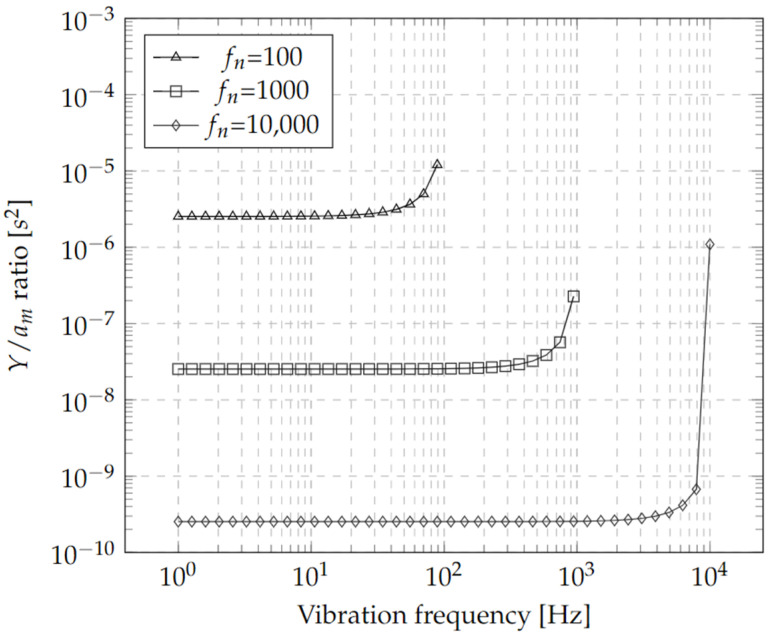
Displacement and Acceleration Ratio vs. Vibration Frequency for Three Different Natural Frequencies *f*_*n*_ = *ω*_*n*_/2*π*.

Figure 4 shows a sweep of the $Y/a_{m}$ ratio, considering three natural frequencies as examples. There is a design trade-off between oscillation amplitude and frequency (*f_n_*), as can be observed in all hypothetical cases considered (*f_n_* =100, 1000 and 10,000 Hz), only as a reference for the selection of the design bandwidth. From this response, the required sensitivity of the optical detector to detect vibration amplitudes can be determined, as well as the frequency bandwidth, as will be described later (Section 2.1.2). Additionally, note that the $Y/a_{m}$ ratio increases abruptly as the vibration frequency ω approaches the resonance frequency $\omega_{n}$. This is the case for the three frequency examples, since the relation $1/\omega_{n}^{2}-\omega^{2}$ is undefined for $\omega =\omega_{n}$.

#### 2.1.2. Optical Detection

To determine the behavior of the optical detection, a sinusoidal wave was considered. The amplitude can be expressed in terms of its initial position *X*_0_, and a change in this position Δ*X* as a function of this sinusoidal vibration is as follows:

(17) $$X=X_{0}+\Delta X$$

(18) $$X=X_{0}+\frac{\lambda_{0}}{4n}\sin\left(\omega_{s}+\varphi \right)$$

where *n* is the refractive index of the cavity (*n* ≈ 1 for air), *λ*_0_ is the wavelength of the light, *ω*_*s*_ is the assumed vibrational frequency of the accelerometer, when a displacement change is applied, and *φ* is the initial phase shift.

As the amplitude *X* can also be understood as a change in cavity length, the difference in phase of the reflected light (*δ*) due to this change is expressed as:

(19) $$\delta =\frac{2\pi nX}{\lambda_{0}}=\frac{2\pi n}{\lambda_{0}}\left(X_{0}+\frac{\lambda_{0}}{4n}\sin\left(\omega_{s}+\varphi \right)\right)$$

It is desirable that the phase difference be limited to the range of 0 ≤ *δ* < 2*π* to avoid equal responses for different amplitude values, so the maximum amplitude of the microstructure was designed to meet the following condition:

(20) $$0<\frac{X}{\lambda_{0}}<1$$

With this condition, for example, if a light source in the infrared wavelength is used (*λ*_0_ = 1 µm), the maximum vibration amplitude of the sensor will be *X*_*max*_ = 0.5 µm. For a length in green (*λ*_0_ = 500 nm), the amplitude would be *X*_*max*_ = 250 nm.

#### 2.1.3. Silicon Microstructure Design

The design of the microstructure was carried out considering the following specifications:

Area of the microdevices should be in the range of 4–25 mm^2^ to allow reflection of a laser with a spot diameter of 1 mm or less. For a ratio area/spot diameter, larger values provide a better tolerance for the laser positioning. This fact provides the first design criterion for the size and shape of the seismic mass of the accelerometer.

Monocrystalline silicon was chosen as the material for the accelerometer to obtain anisotropic (orientation or direction dependent) mechanical properties and low-temperature variations. In [27], measurements of temperature variation can be found, differentiating between the three main crystallographic directions (<100>, <110> and <111>) to which mechanical stress is applied. Together, these two features optimize the stability of the sensor and its insensitivity to vibration components in other axes when selecting the bending direction of the microstructure springs. In this case, the <100> direction was selected.

An optical detection, and the use of silicon, are of interest for a vibration sensor implementation in a temperature environment where conventional electronic components might prematurely fail. This upper limit is set by the application grade of the component, which usually is 70 °C for commercial, 85 °C for industrial and 125 °C for military. Since most device manufacturers define their own grades, these ranges are not standardized, and above industrial grade there is a limitation in the market for the diversity and availability of electronic components. Therefore, the 85 °C specification is selected as a reference for material and assembly process definition. Additionally, optical detection is immune to electromagnetic and radio frequency interferences.

**Figure 5:**
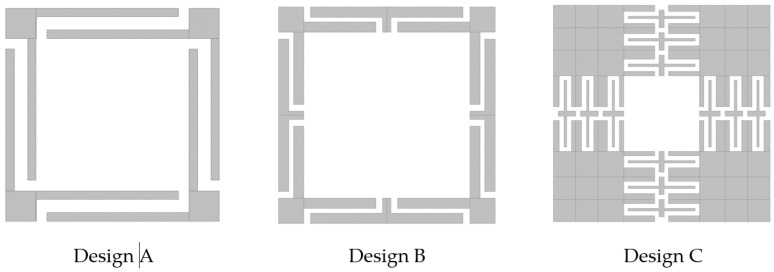
Geometries of accelerometers designed to meet the established specifications, with (**a**) Z, (**b**) double Z, and (**c**) a series of three folded beam arrangements.

From Equation (20), vibration amplitudes of ±1 μm were considered for its use in the range of visible and infrared wavelengths. Additionally, with the aid of the Figure 5, the operation frequency of the accelerometer can be determined, with a bandwidth lower than 2 KHz.

From the last point, the first objective was to design a microstructure that has a vibration amplitude of ±1 μm, at frequencies lower than 2 kHz, for use with wavelengths between visible and infrared. For this purpose, three silicon microstructure geometries shown in Figure 5 were designed on the base of [25,28,29] and the system requirements. They were dimensioned and evaluated by Finite Element Analysis (FEA) to estimate their performance. The movement of the mass shall be predominantly out of plane to separate the first resonant mode from higher modes that produce unstable oscillation or introduce measurement uncertainty effects.

### 2.2. Finite Element Analysis

#### Modelling

**Figure 6:**
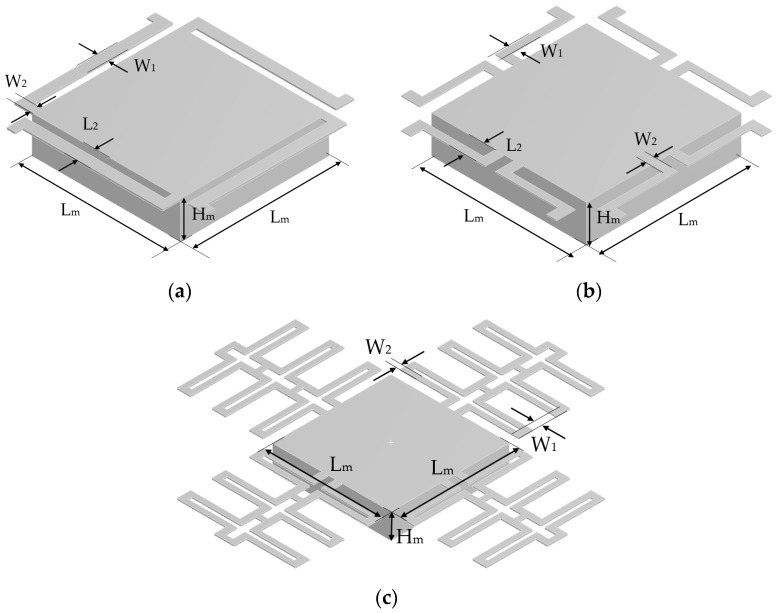
CAD Model of (**a**) Design A, (**b**) Design B, and (**c**) Design C.

The models, optimized by FEA using parameterization, are shown in Figure 6. Their dimensions are specified in Table 1.

For the design analysis, three main studies were carried out:

i. A steady-state study to verify the maximum displacement amplitude at a given acceleration and the static effects, mainly the sensitivity to gravitational attraction as a function of the orientation of the microstructure with respect to the Earth (Section 2.3.1).
ii. A modal study to determine the natural resonance modes and their frequencies is performed in Section 2.3.2.
iii. A harmonic study to find the frequency response of the microstructure subjected to vibrations at a known frequency (Section 2.3.3).

### 2.3. Simulation Results

#### 2.3.1. Steady-State Study

**Figure 7:**
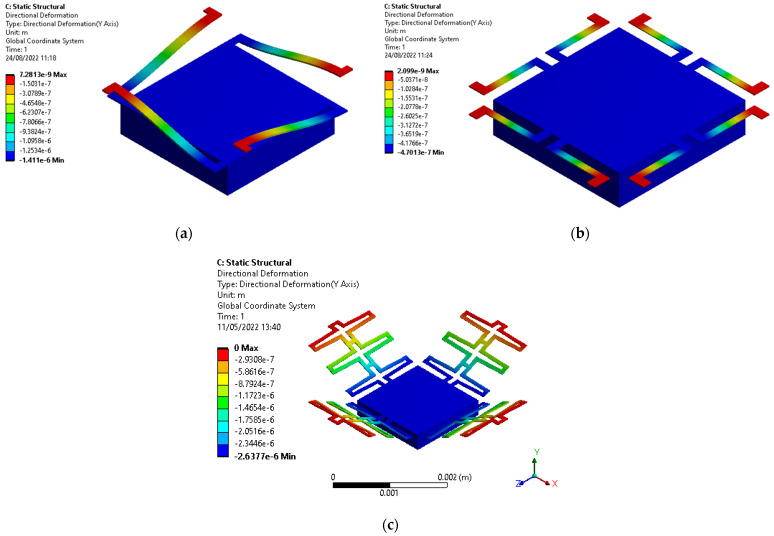
Steady-state FEA of designs A, B and C subjected to a static Acceleration of 10 g. (**a**) Design A with 1.41 µm mass displacement. (**b**) Design B with 0.47 µm mass displacement. (**c**) Design C with 2.64 µm mass displacement.

As an example, Figure 7 shows the result of a static analysis of Accelerometer Design C, subjected to a constant acceleration of 10 g, where a maximum amplitude of 2.637 µm was obtained. In Table 2, technical details of the FEA are given. This analysis was used with a sweep for several accelerations, and together with a linear approximation, the ratio change of 140 nm/g, 47 nm/g and 53 nm/g for designs A, B, and C was obtained.

#### 2.3.2. Modal Analysis

**Figure 8:**
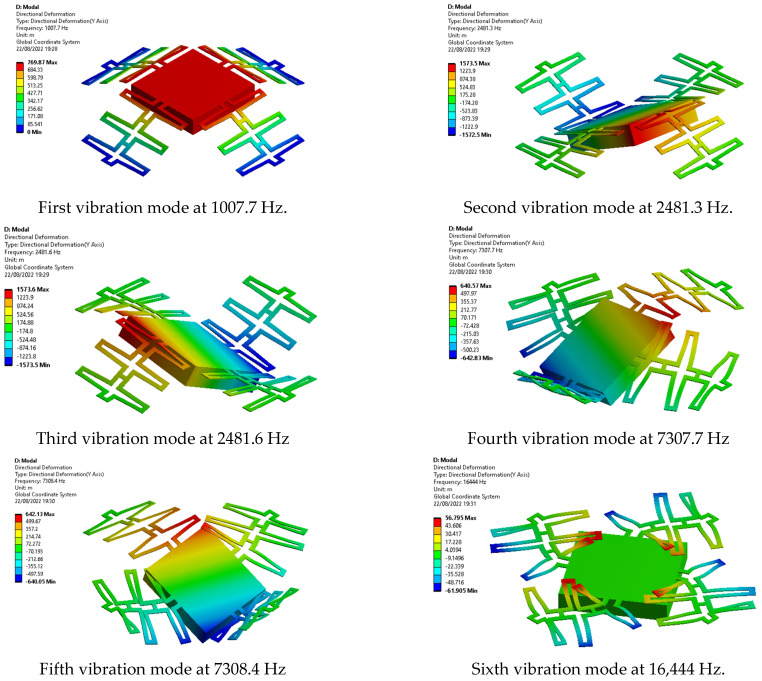
Modal frequencies for Design C.

In Figure 8, the first six modal frequencies are shown, and it can be observed that the modal response of interest is the first one of 1007.7 Hz.

Table 3 shows the results of the frequencies of the first six modes of vibration for the three designs considered, where Design C shows the lowest vibration frequencies.

#### 2.3.3. Harmonic Analysis

**Figure 9:**
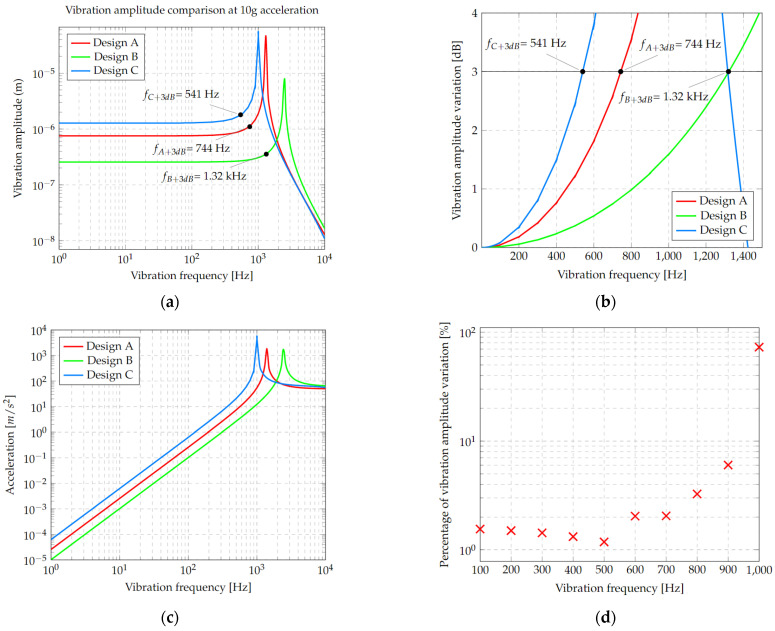
(**a**) Vibration amplitude of the three designs subjected to an acceleration of 10 g relative to the vibration frequency. Data are shown in a logarithmic scale. (**b**) Frequency bandwidth for A, B and C designs using a maximum vibration amplitude variation of +3 dB from the flat response. (**c**) Proof mass acceleration in relation to vibration frequency for the three designs. (**d**) Percentage of vibration amplitude variation between 25 °C and 200 °C temperature for design C.

The results of the harmonic analysis, using an acceleration of 10 g, are shown in Figure 9; these trends are consistent with the graph shown in Figure 4. It can be observed that the accelerometer with the lowest vibration frequency and the highest amplitude corresponds again to design C (Figure 9a). In addition, it can be observed that if the bandwidth decreases, the oscillation amplitude is greater, maintaining in all cases this design trade-off.

From the latter analysis, the operating range was defined in accordance with the standard criteria, choosing the points where the amplitude frequency response would be less than +3 dB (Figure 9b). These results were in DC ranges of 744 Hz for design A, at 1.32 kHz for design B, and at 541 Hz for design C.

The acceleration response of the microstructure was estimated using the results of the harmonic analysis (Figure 9c) corresponding to the acceleration at one point on the reflecting surface. In this case, a similar behavior is observed, which is expected in concordance with the design guides used.

The maximum operating cycles for the microstructure were estimated using a simulation of the first principal stress and were compared with the experimental results reported for silicon [30].

For the structures under analysis, the maximum stress was less than 240 MPa, which exceeds 1010 cycles of life according to [30]. The maximum stress values obtained are under the ultimate stress of silicon. However, these results are preliminary in terms of determining the microstructure design and not the entire probe. They are only valid for a single axis normal to the surface and are used for the proof-of-concept. From these results, design C was chosen due to the following facts:

- Its vibration amplitude is the largest, reaching a displacement of 1 μm at 10 g, which will allow the performance of the optical detection system to be evaluated.
- It has its second vibration mode of 2481 Hz further away from the frequency of the first one of 1007 Hz, in comparison with other designs, which allows more stability in the experimental test range.
- The seismic mass can be adjusted to various dimensions, and even consider circular shapes.

An analysis regarding the operating temperature was also performed using data from experimental measurements of Young’s modulus as a function of temperature and considering different layer thicknesses, as reported by [31]. Tests were carried out for each of the designs. In Figure 9d, the results obtained with accelerometer C are given, where no significant variations are observed when the analysis is carried out at 25 °C and 200 °C. Furthermore, an increasing behavior in the amplitude can be observed for frequencies between 0 and 200 Hz, up to a maximum of 80 µm, and then rapidly decaying. This amplitude is useful to determine the working range, considering 200 °C as the limit.

### 2.4. Vibration Sensor Package Design

**Figure 10:**
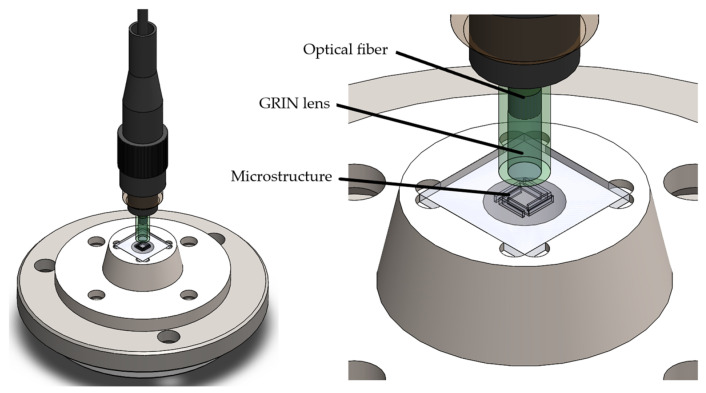
Final Housing Design with Incorporation of GRIN Lens.

For the design of the probe housing, only the mounting specifications (probe mass and shaker adaptation chords) are required to be fulfilled. However, additional elements focused on the future integration of optic fiber were considered. The conceptual design is shown in Figure 10, which is formed by two parts:

(a) A support for the microstructure (in green)
(b) The encapsulation made in Steel SAE 304 (in metallic color)

These two parts are designed to form a conical assembly that enables a reduction in the misalignment effect between the microstructure and the incident light beam.

The housing already incorporates an adaptation for the future fiber-optic connection (Figure 10). This connection foresees the use of the probe with an optic fiber-constructed interferometer, which will be suitable for testing in a relevant environment. This connection will be made by means of a ferrule (Figure 10) to fix the fiber core by means of high-temperature epoxy resin.

To mitigate the effect of a power reduction due to misalignment, the integration of a Gradient Refractive Index (GRIN) lens of concentration of the reflected beam was considered. This type of lens presents a gradient in the refractive index, which is used to facilitate the interface of collimated light and the optic fiber.

### 2.5. Test Bench Design

#### 2.5.1. Interferometer Scheme

**Figure 11:**
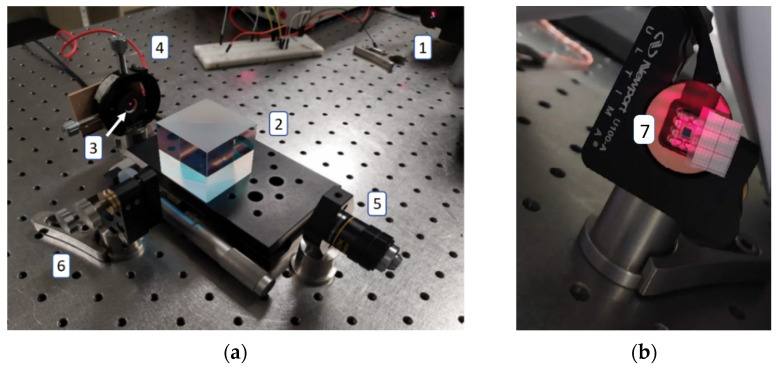
(**a**) Microstructure Test Bench Design, (**b**) Interferogram Projected from Test Bench on (7), the Selected Photodiode, which is aligned with (5), the Projection Objective.

The general scheme of the test bench design (Figure 1) is shown in Figure 11, considering the following components, which were installed on an anti-vibration table:

1. Light source: Linearly polarized He-Ne laser, with wavelength of 633 nm, power of 2 mW, divergence of 1.3 mrad, and polarization ratio of 500:1.
2. Beam splitter: 50:50 in non-polarizing cube in the range of 400 to 700 nm.
3. Microestructure mirror: Monocrystalline silicon microstructure under test
4. Mechanical support: Two-degree-of-freedom circular mirror assembly and 8.3 mrad/rev. resolution adjustment.
5. Projection objective: With 10× magnification and 0.25 aperture
6. Reference mirror: Dielectric fused silica mirror with reflective coating for a 400 to 700 nm range.
7. Photodiode: With integrated preamplifier OPT101, detection area of 2.29 mm × 2.29 mm, sensitivity of 0.45 A/W (at 650 nm), and bandwidth of 14 kHz.

#### 2.5.2. Photodiode Response

For the estimation of the photodiode response, due to the projection of the inter-ferometer, a calculation was performed using an algorithm code in Octave (see Appendix A). This calculation involved estimating the area of exposure to incident light, considering perfectly defined boundaries, on the surface of the photodiode.

The code developed uses the initial considerations given by the fringe size of the interferometer projection, the size of the active area of the photodiode and tilt angle α between them. Using these data, the algorithm generates polygons, represented by a two-column matrix containing *x* and *y* coordinates of its vertex, for the rectangular fringes and the rectangular photodiode. Then, the sensor polygon matrix *S_Ph_* is rotated by an angle $\theta_{Ph}$, to model non-ideal alignment with the fringes. The rotation is performed using a rotation matrix in Euclidean space as follows:

(21) $$RS_{Ph}=SPh*\left[\begin{array}{cc} cos\theta_{Ph} & -sin\theta_{Ph}\\ sin\theta_{Ph} & cos\theta_{Ph} \end{array}\right]$$

where $RS_{Ph}$ is the resultant rotated matrix. Then, a new polygon is generated by the function *oc_polybool* contained in the *octclip* package for Octave. This function performs a Boolean AND operation between the light fringes and rotated photodiode area polygons, using the Greiner–Hormann algorithm, to obtain matrixes for light-exposed areas on the photodiode. Subsequently, the *polyarea* function is used to calculate the exposed area by the triangle method. This operation is repeated for a sweep of the fringe position, simulating a drift *y_f_* due to a linear change in position of the microstructure.

**Figure 12:**
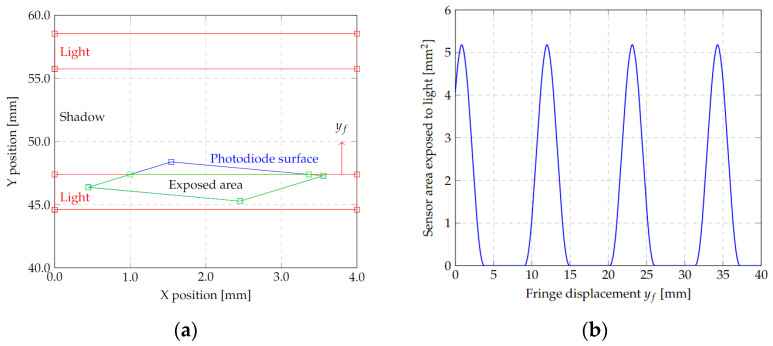
(**a**) Geometric reconstruction of the alignment of photodiode and interferogram. It shows the fringes (red), the photodiode area with an angle *φ* from the fringe (blue) and the area exposed to incident light of the interferogram fringes (green). (**b**) Estimated exposed area change by fringe displacement *y_f,_* which can be approximated by a sinusoidal function.

The results obtained, shown in Figure 12, were mainly used for the test bench setup to calculate the transfer function from the fringe displacement, due to the microsensor vibration and the change in the photodiode exposed area, which can be numerically approximated by a rectified sinusoidal function of the form:

(22) $$V_{ph}\left(x\right)=\{\begin{array}{cc} A_{v}sin\left(\omega_{ph}y_{f}+\varphi \right)+x_{off} & A_{v}sin\left(\omega_{ph}y_{f}+\varphi \right)+x_{off}\geq 0\\ 0 & A_{v}sin\left(\omega_{ph}y_{f}+\varphi \right)+x_{off}<0 \end{array}$$

where $V_{ph}$ is the photodiode output voltage function due to the fringe displacement $y_{f}$, $A_{v}$ is the voltage amplitude, $\omega_{ph}$ is the frequency of fringe movement, *φ*; is the photodiode tilt angle and $x_{off}$ is the correction factor for the initial position of the fringe.

The rectification effect can be eliminated if the fringes are the same width than the photodiode, or its value will differ from zero if photodiode is exposed to two fringes at the same time. Therefore, it is desired to have a fringe aligned with the photodiode and change the projection objective distance to adjust the fringe width to the photodiode area. For this case, ideally, $V_{ph}\left(x\right)=A_{v}sin\left(\omega_{ph}y_{f}+\varphi \right)+x_{off}$.

## 3. Manufacturing

### 3.1. Manufacture of Accelerometers

**Figure 13:**
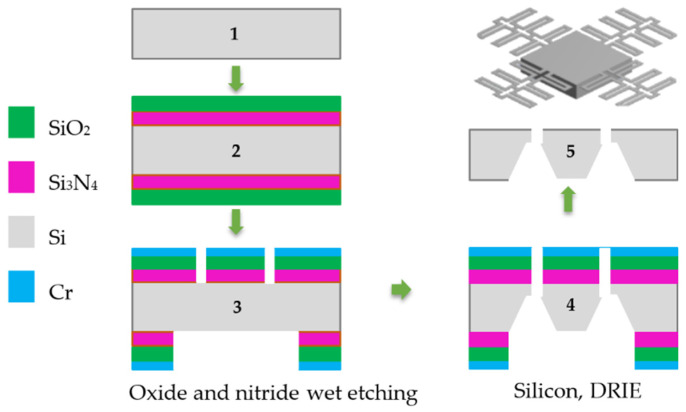
Microstructure Manufacturing Process.

The manufacture process of silicon microstructures is shown in Figure 13, where a sequence of cross-sections is presented to visualize the attack and deposition stages. This sequence is described below:

i. A 100 mm diameter, 400 μm-thick monocrystalline silicon wafer with a polished top side (with a thickness roughness less than 10 nm) and <100> crystalline orientation is used.
ii. Silicon nitride (300 nm) and silicon oxide (1.5 μm) layers are deposited through a sputtering process on both faces. Their roughness is not relevant because these layers will be used as sacrificial material.
iii. A pattern is etched on the oxide and nitride layers of both faces by the next steps:
  a. A chromium deposit (300 nm) is made by electron-beam deposition
  b. By means of a photolithography process, a pattern is etched on the chromium with wet etching by chrome etchant.
  c. The oxide pattern is etched with hydrofluoric acid. The chromium acts as a protective layer for the regions not to be etched, until the silicon is exposed.
iv. Deep reactive ion etching (DRIE) is performed on both sides of the wafer, one after the other. The back side is etched first; the duration of the etching sets the proof mass thickness of the microstructure. Subsequently, the pattern of the beam supports is etched on the top (polished) side until it is transferred to the cavity formed on the bottom side.
v. The sacrificial layers, made of nitride, oxide, chromium, and photoresist residues, were removed. For the sake of simplicity, the photo resin process is not shown. The liberated microstructure was obtained.

**Figure 14:**
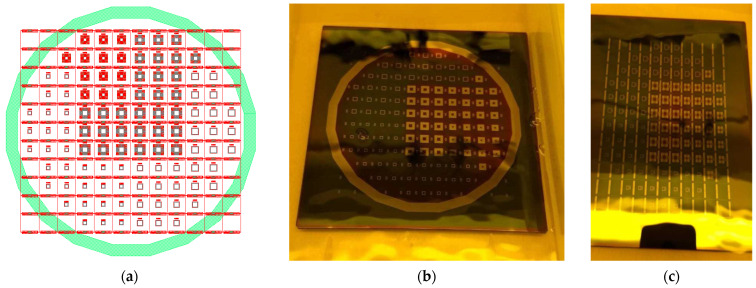
(**a**) Mask Design for the Entire 400 mm Wafer. (**b**) Lower and (**c**) Upper Photolithographic Masks.

For the manufacturing process of the three designs, obtained by FEA, the micromanufacture mask files were also made. These designs are shown in Figure 14. They consist of four layers bonded together to form the two required masks (top and bottom).

The masks consist of a pattern of the three designs with several repeats. Each pattern has a spacing of approximately 1.22 mm to allow the chip dicing operation to be carried out without damaging any microstructure. A total of 104 chips can be manufactured on a single wafer with a single process. The design of the complete wafer is shown in Figure 14a.

The masks and devices were manufactured in CIDESI’s Micro-technologies Laboratory. The masks manufactured are shown in Figure 14b,c. They can be used as a master pattern and reused in various manufacturing processes. They consist of a 5-inch × 5-inch soda lime substrate with a chromium layer that is etched to open windows to allow selective exposure of UV radiation to the photoresist layer, which is deposited on the wafer.

**Figure 15:**
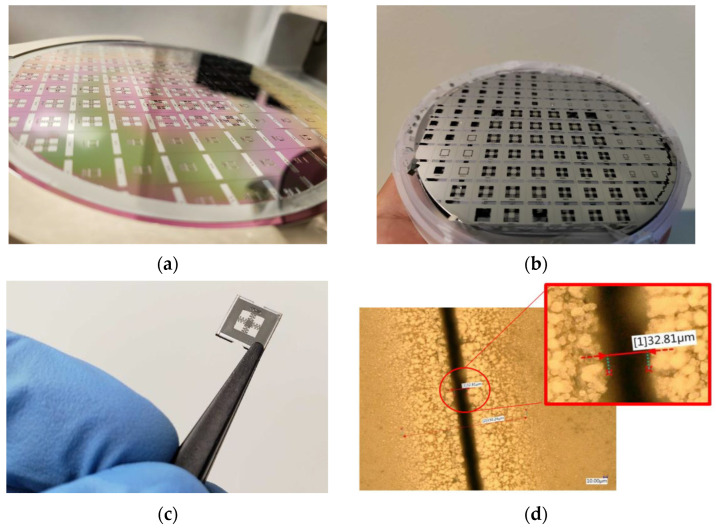
Wafer during manufacturing. (**a**) Wafer during manufacturing (step #4). (**b**) Wafer after release of sacrificial layers in (step #5). (**c**) Single chip after dicing using laser ablation. (**d**) Zoom in of the groove produced by laser ablation.

The wafer transformed during the manufacturing process is shown in Figure 15. After releasing the microstructures, chip dicing is carried out by laser ablation with a focus spot size of approximately 25 μm in diameter. This produces a 33 μm groove with thermal damage extending to 330 μm, as shown in Figure 15d.

A total of 71 devices passed visual inspection and dimensional characterization; 28 correspond to design A, 4 to design B, and 39 to design C. This is mainly due to DRIE etching, where a sacrificial wafer is used as the base. These two wafers must be separated manually, which resulted in damage in the first iterations of manufacturing. Because the focus is on microstructures with lower frequency response, the largest number of design C devices were fabricated.

### 3.2. Vibration Measurement Probe Housing

**Figure 16:**
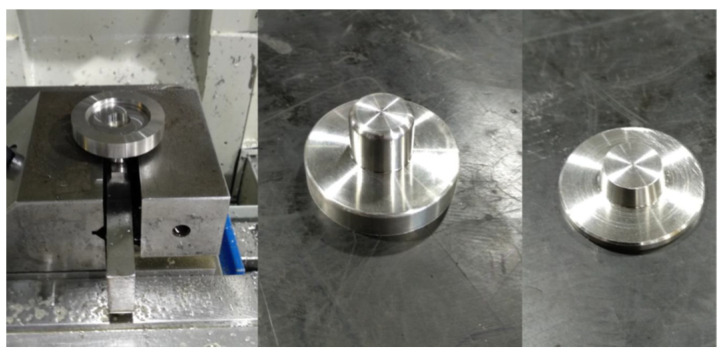
Manufacture of package.

The encapsulation was fabricated at CIDESI, using numerically controlled machines and SAE304 stainless steel (Figure 16). This design yielded a probe that when assembled has a total mass of 158 g and a controlled gap between the microstructure surface and the GRIN lens of 1.14 mm.

**Figure 17:**
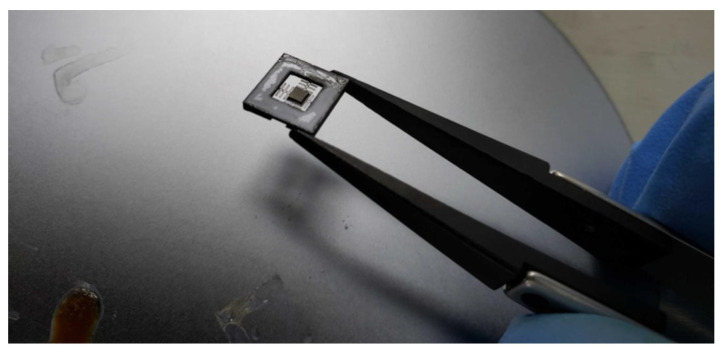
Adhesion Process of the Microstructure to the Probe Support.

The microstructure was adhered to the support (Figure 17) using ADH-119FO epoxy resin of low viscosity and high temperature, which operates up to 250 °C. Due to its refractive index, this resin will also be used in the sealing of fiber optic ferrules.

### 3.3. Implementation of the Test Bench

**Figure 18:**
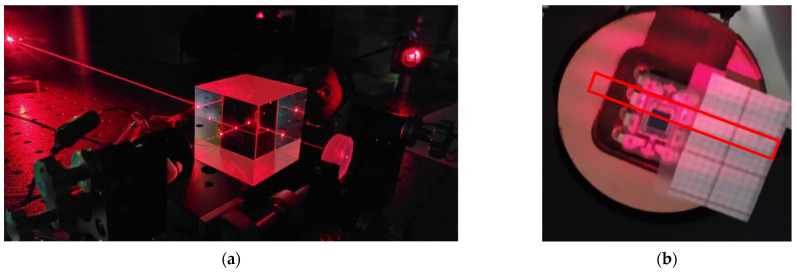
(**a**) Test Bench with Michelson Interferometer. (**b**) Interferogram to Photodetector Projection Adjustment Showing the Match between the Size and Angle of the Fringe and the Area of the Photodetector.

Two different tests were performed mainly for the evaluation of the detection system and for the analysis of the microstructure. Both use the same designed optical detection scheme (Figure 18).

The first step in the assembly is alignment. By means of the mirror supports, the laser, and the placement of the beam splitter, the incident rays were aligned on both surfaces, generating their interference. This interference was subsequently aligned with the purpose of projecting it on the photodetector.

**Figure 19:**
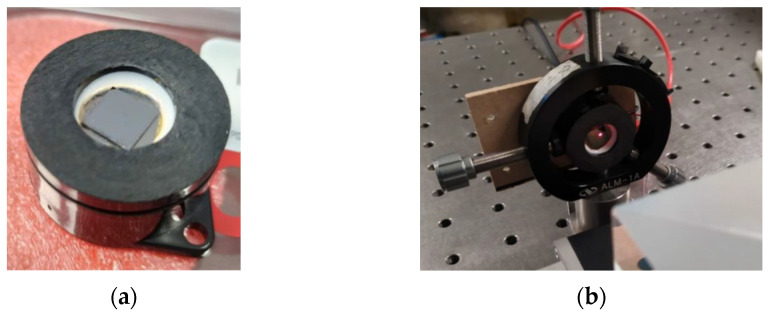
Test of Optical Detection System with Piezoelectric Actuator. (**a**) Silicon Substrate on Piezoelectric Actuator. (**b**) Alignment of Silicon Substrate on Piezoelectric Actuator with Interferometer Laser.

Since the photodetector is sensitive to visible light, the tests were performed predominantly without ambient illumination. For initial alignment and testing of the detection system, a monocrystalline silicon substrate attached to a membrane actuated by a piezoelectric buzzer was used, as shown in Figure 19a.

Subsequently, the projection on the photodetector was aligned, adjusting the position and separation between the target and the photodetector. This way, it is possible to achieve the projection of fringes that coincide in angle and size with the area of the photodetector (Figure 19b). This alignment operation is facilitated by manufacturing a circular PCB board with the same diameter as the mirrors to use the two-degree-of-freedom alignment supports.

**Figure 20:**
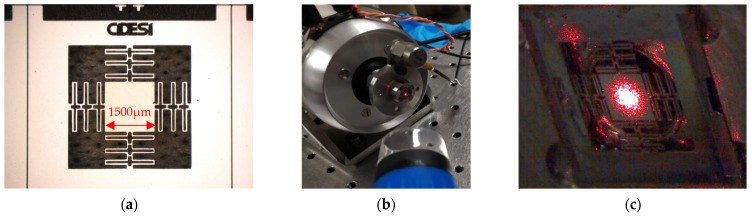
(**a**) Microphotography of fabricated microstructure on wafer. (**b**) Mount assembly with attached microstructure, reference accelerometer and alignment camera. (**c**) View of laser spot alignment on microstructure.

A final test setup was then performed using a shaker, where the fabricated microstructure (a) was placed using its steel support (without the encapsulation or protective jacket to facilitate the placement of the incident laser beam). In this support, an adaptation was manufactured to place a reference accelerometer, as shown in Figure 20. The shaker can operate in a frequency range from 5 Hz up to 13 kHz with a maximum acceleration of 91 g for a mass of 190 gr.

To check the integrity of the assembly, the microstructure attached to the conical support (Figure 20b) was checked using a digital optical microscope (Figure 20c). Thus, measurements of additional geometrical dimensions were also performed. The data obtained were used for experimental testing.

**Figure 21:**
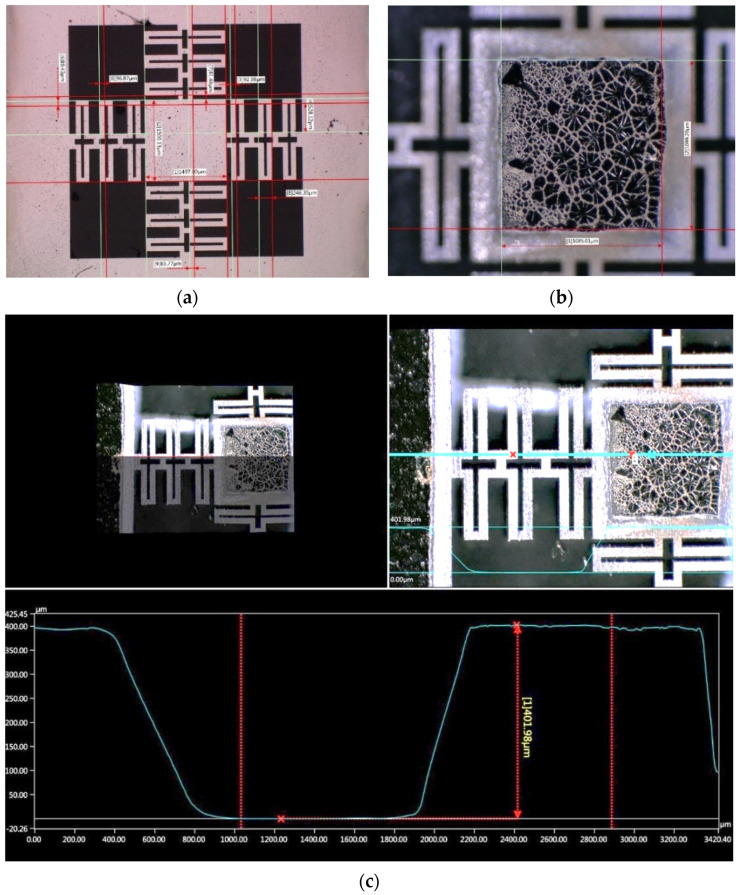
Measurement of microstructure with a digital optical microscope. (**a**) Microstructure dimensions measurement. (**b**) Measurement of bottom face reduction of proof mass (**c**) Top and left: 3D recreation model, cross-section view, using an optical profiler. Top and right: Cross-section line (blue) for height measurement. Bottom: Height profile corresponding to the blue line in the top and right image.

**Figure 22:**
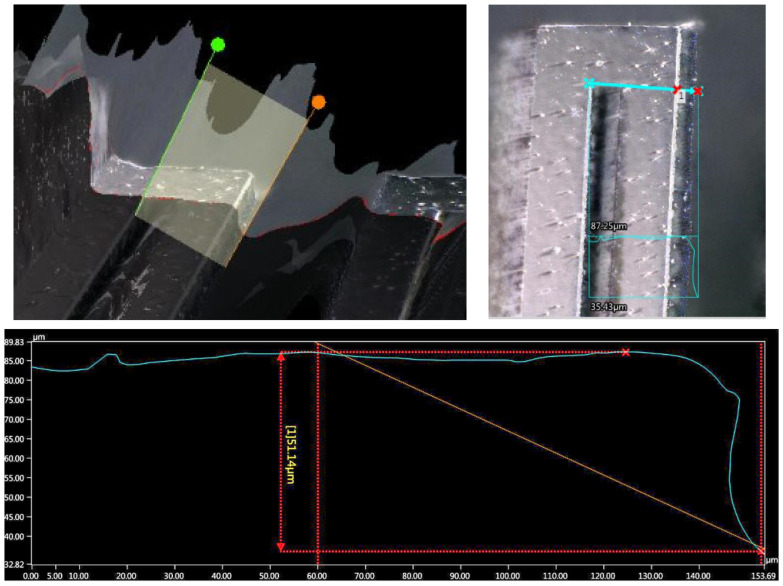
Measurement of beam thickness.

In Figure 21, the measurement of the accelerometer cross-section profile of Design C is shown by using an optical microscope VHX-6000. The measurement of the beam cross-section was not considered with a mechanical profiler due to the risk of damaging the integrity of the structure and the equipment. Therefore, a measurement with the VHX-6000 in a tilted configuration (60°) is shown in Figure 22. With this condition, the beam thickness was estimated using a software tilt correction (brown line in bottom plot of Figure 22). A comparison of geometric sizes is given in Table 4.

From Table 4, it can be observed how the highest error corresponds to the mass and beam thickness. This is a consequence of the limitations in the silicon double-etching process, which lacks a stop layer, or a similar thickness etching control, for example, by using a SOI substrate. In contrast, the described process (Section 3.1) is selected as it alleviates additional specifications, useful in technology transfer, such as material and equipment availability, and cost reduction.

**Figure 23:**
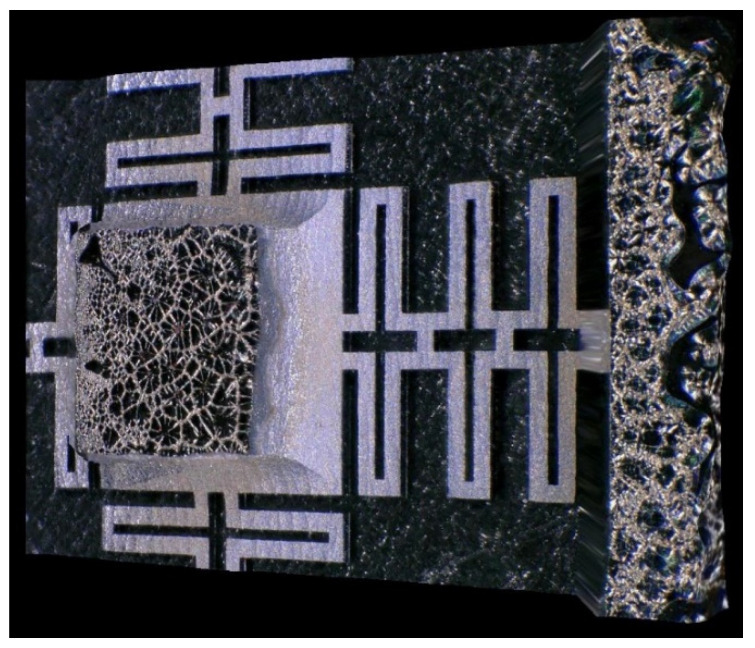
Microphotograph of microstructure bottom view with an angle of 60°. The variation in proof mass perimeter due to fabrication process is shown.

For the proof mass, the main error is an outcome from the lateral etching, which can be observed in Figure 23. However, once characterized, the mass reduction can be compensated with a change in the layout dimensions.

Both proof mass and beam thickness variation have an important impact on the behavior of the device. From the analytical modeling in Section 2.1.1, it is known that the beam thickness changes the stiffness coefficient of the vibration model and the mass impacts in the vibration bandwidth.

## 4. Experimental Tests

### 4.1. Optical System Evaluation

**Figure 24:**
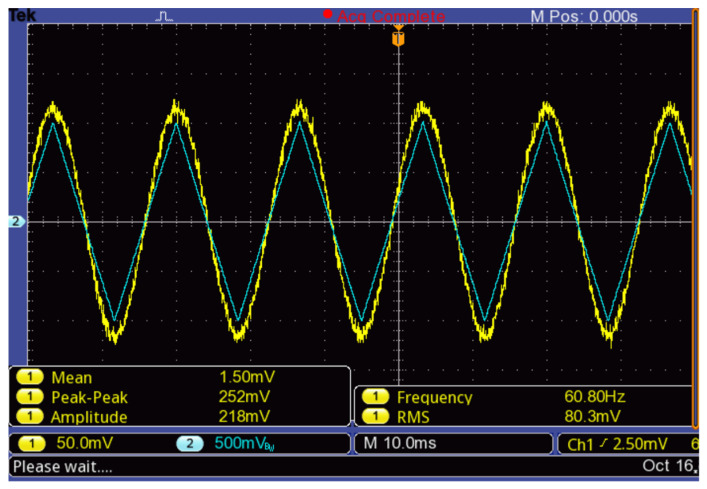
Photodetector Signal with System Aligned to Silicon Substrate Actuated by a Piezoelectric Buzzer.

The optical system was evaluated using, first, the test setup with the piezoelectric actuator with a polished silicon substrate attached on top of the piezoelectric actuator (Figure 19). The actuator was connected to a signal generator Tektronix AFG1022 to monitor the deformation, which is given by the displacement of the central area at the normal direction to the surface and was measured in steady state at a sensitivity rate of *S_piezo_* = 0.2 μm/V (Figure 24). This measurement was obtained by measuring the focusing distance change in the silicon surface over the piezoelectric for different voltage stimulus with a Keyence VHX6000 digital microscope.

The vibration amplitude with the piezoelectric actuator is not constant at different frequencies, but for signals below 60 Hz, it remains very close to 0.2 μm/V. The polarized photodetector was connected to an oscilloscope Tektronix TBS1022 with a bandwidth limited to 10 MHz. The signal obtained (in yellow) by applying a triangular signal (in green) on the substrate was measured with an oscilloscope (Figure 24).

The following points can be established from the results of this test:

1. The polished monocrystalline silicon substrate has optical properties in the visible wavelength range, which is sufficient for use in an interferometric vibration detection scheme.
2. The fringe shift coincides with the ratio of λ/2 = 316.5 nm. Consequently, from Figure 24, where the amplitude of the applied triangular signal is *A_piezo_* = 1 V, the vibration generated has an amplitude of ±*A_piezo_*∙*S_piezo_* = ±200 nm.
3. From the photodetector response to the measured deflection amplitude of ±200 nm, the sensitivity of the entire system to the sensor can be calculated by a linear approximation as *S_sys_* = 200 nm/0.115 V = 1.74 μm/V.
4. The optical system cannot be used to measure signals with frequencies that are higher than 14 kHz due to the bandwidth of the photodetector selected.
5. To measure the relative position of the microstructure, the measurement system must have a vibration in phase with its reference frame.
6. With this setup, a peak-to-peak noise voltage ($V_{n}$) lower than 10 mV is achieved without further signal processing. Therefore, vibration resolution can be calculated using the linear sensitivity approximation as:

(23) $$Y_{fmin}=S_{sys}V_{n}=17.5\;nm$$

This resolution might be improved in the future by including signal filtering, noise reduction techniques and a photodiode with higher SNR specifications.

**Figure 25:**
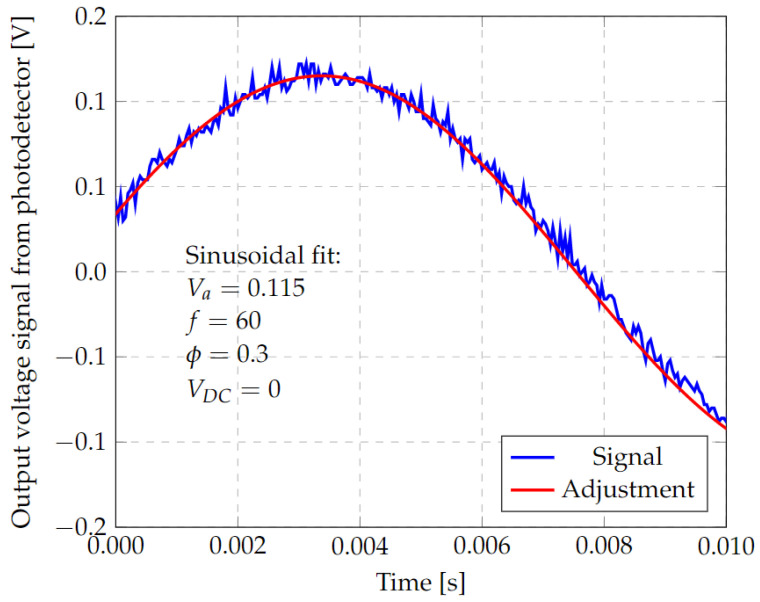
Approximation of the photodetector response to the ideal shape by an area calculation algorithm.

The results in Figure 24 have been fitted, as described in Section 2.5.2, to a sinusoidal function of the form $V_{ph}\left(x\right)=A_{v}sin\left(\omega_{ph}y_{f}+\varphi \right)+x_{off}$. The fitting obtained is shown in Figure 25 where $A_{v}=0.115V$, $\omega_{ph}/2\pi$ = 60 Hz, φ=0.3 rad and $x_{off}=0$. In consequence, this setup can be used to calibrate the photodiode alignment to obtain a minimum value of *φ*.

### 4.2. Microstructure Performance Test

**Figure 26:**
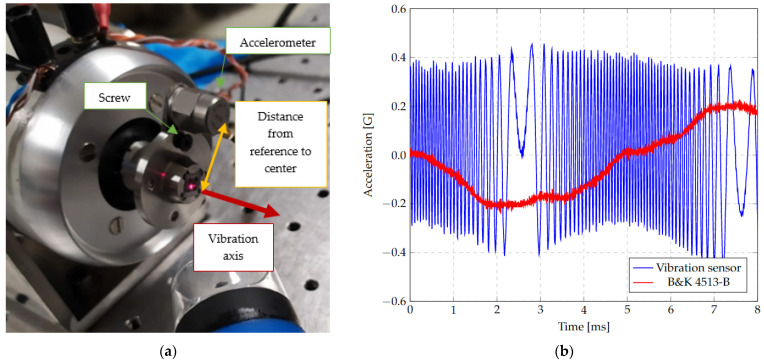
(**a**) Assembly of Microstructure on Test Bench. (**b**) Output Waveforms from Vibration Sensor Design C and the Reference Accelerometer (B&K 4513-B). The waveforms are obtained with a 100 Hz and 0.2 g vibration.

To evaluate the accelerometer, it was mounted on the shaker and aligned with the interferometer (Figure 26a). In this case, the output of the photodetector was connected directly to the shaker driver (VR9500) together with the reference accelerometer. The accelerometer is a B&K 4513-B. The Vibration View 2017 control software was also used. The test results are shown in Figure 26b. Afterwards, repetitions were performed for different accelerations and vibration frequencies, and the following observations were obtained:

1. There is a small distortion of the reference accelerometer signal due to the adaptation of the accelerometer manufactured, which is not concentric and is made by means of a screw that does not ideally approximate a rigid solid. The effect is negligible and can be reduced by placing the accelerometer concentrically to the microstructure support for future evaluations.
2. To obtain the vibration signal of the microstructure with reference to its local coordinate system, it is required that the interferometer be vibrating next to the reference of the support to avoid the modification of the trajectory with the main vibration. Therefore, the result of this test corresponds to a composite signal between the vibration of the support plus the vibration of the microstructure.
3. Because of the previous point, there is a limitation on the spectrum and amplitude that can be measured, because to produce a significant vibration in the microstructure, a minimum acceleration, which depends on the used frequency, is required. This acceleration cannot be increased arbitrarily, since if the total amplitude (of the support plus the microstructure) exceeds the maximum range and the change of fringes produces a saturation of peaks in the response, it is not possible to distinguish between them.

A frequency modulation in phase with the amplitude peaks of the vibration can be observed, allowing the frequency of the vibration sensor to be calculated. The number of peaks between these states is related to the amplitude of the vibration of the reference sensor.

The response of this test can be defined as the sum of two functions referring to the support frame of the microstructure.

(24) $$\gamma =\gamma_{sh}+\gamma_{s}$$

where $\gamma_{sh}=A_{sh}sin\left(\omega_{sh}t+\varphi_{sh}\right)$, and $\gamma_{s}=A_{s}sin\left(\omega_{sh}t+\varphi_{sh}+\pi \right)$.

*γ*_*sh*_ is the shaker’s sinusoidal vibration with amplitude *a*_*sh*_, angular frequency *ω*_*sh*_ and phase *φ*_*sh*_, and *γ*_*s*_ is the sinusoidal vibration of the sensor with amplitude *a*_*s*_, angular frequency *ω*_*sh*_ and phase *φ*_*sh*__+__*π*_. The vibration of the microstructure has the same frequency as the excitation but is out-of-phase by *π* radians. Therefore, applying trigonometric identities:

(25) $$\gamma =(A_{sh}-A_{s})sin\left(\omega_{sh}t+\varphi_{sh}\right)$$

The voltage response of the photodiode (*V*_*ph*_), considering the area coverage analysis is given by:

(26) $$V_{ph}\left(x\right)=A_{v}sin\left(\omega_{ph}x+\varphi \right)+x_{off}$$

where *x* is the displacement of the microstructure, $A_{v}$ is the amplitude of the electrical signal, *φ* is its phase and *x*_*off*_ is the DC offset voltage of the signal. Then, the measured response of the photodiode *V*_*ph*_(𝛾(𝑡)) is given by:

(27) $$V_{ph}\left(\gamma \left(t\right)\right)=A_{v}sin\left(\omega_{ph}\gamma \left(t\right)+\varphi \right)+x_{off}$$

Substituting *γ*(*t*) from Equation (25), the expression (27) is obtained:

(28) $$V_{ph}\left(\gamma \left(t\right)\right)=A_{v}sin\left[\left(\omega_{ph}(A_{sh}-A_{s}\right)sin\left(\omega_{sh}t+\varphi_{sh}\right)+\varphi \right]+x_{off}$$

Since the shaker instrumentation can be considered without phase shift and the photodetector offset voltage is negligible, Equation (28) can be reduced to:

(29) $$V_{ph}\left(\gamma \left(t\right)\right)=A_{v}sin\left[(\alpha sin\left(\omega_{sh}t+\varphi_{sh}\right)+\varphi \right]$$

with $\alpha =\omega_{ph}(A_{sh}-A_{s})$, $V_{ph}\left(\gamma \left(t\right)\right)$. Equation (29) describes the signal shape of the device under testing, shown in blue in Figure 26.

On the other hand, a function of the form (*x*) = *sin*(*sin*(*x*)) can be expanded as a Fourier series, using:

(30) $$f\left(x\right)=2\overset{\infty }{\underset{0}{\sum }}J_{\left(2n+1\right)}\left(1\right)\sin\left(\left(2n+1\right)x\right)$$

where *J* is the Bessel function of the first kind. Therefore, neglecting the phase, *V*_*ph*_(*γ*(*t*)) can be expressed approximately by the first three terms of the series:

(31) $$V_{ph}\left(\gamma \left(t\right)\right)=A_{v}\left[0.8801\alpha \sin\left(\omega_{sh}t\right)+0.03911\alpha \sin\left(3\omega_{sh}t\right)+0.0005\;\alpha \sin\left(5\omega_{sh}t\right)\right]$$

Filtering out the frequency components above *ω*_*sh*_, a signal *V*_*ph*__-__*fillt*_ is obtained, which can be expressed as:

(32) $$V_{ph-filt}\approx A_{v}\left[0.8801\alpha \;sin\left(\omega_{sh}t\right)\right]$$

**Figure 27:**
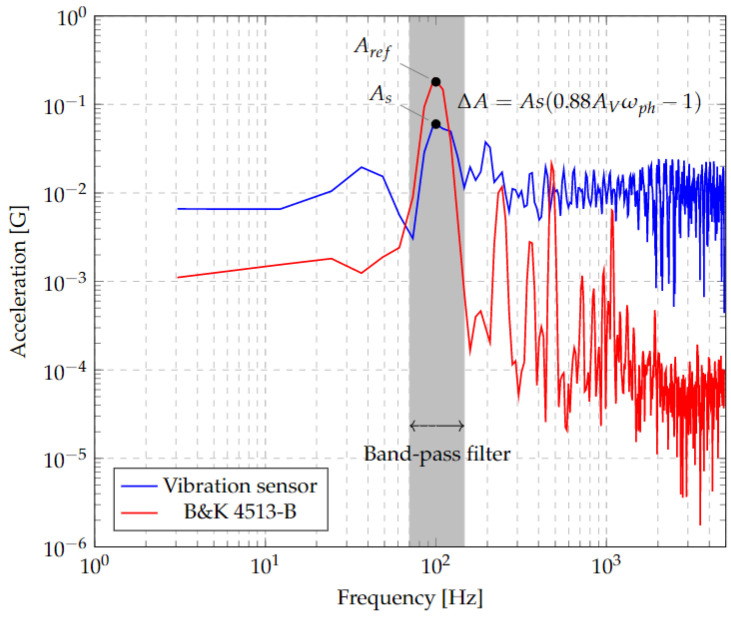
Comparison between Signal Spectra between Vibration Sensor and Reference Accelerometer. The acceleration amplitudes at 100 Hz are labeled and a band pass filter range is shown for comparison.

Substituting the value of *α*, given as part of Equation (29), Equation (33) is obtained:

(33) $$V_{ph-filt}\approx 0.8801A_{v}\omega_{ph}(A_{sh}-A_{s})sin\left(\omega_{sh}t\right)$$

which allows a comparison of the signals from the reference accelerometer and the one obtained from the sensor at the shaker oscillation frequency, *ω*_*sh*_, which for this test corresponds to 100 Hz, as shown by the peak acceleration in Figure 27. Thus, the acceleration amplitude seen in the signal spectrum of the sensor under test is proportional to the acceleration of the reference sensor, given by:

(34) $$a_{ref}=0.88011A_{v}\omega_{ph}A_{s}$$

where *A*_*V*_ has integrated photodiode response information relative to the vibration displacement, any nonlinearity or error, and its frequency sensitivity, which can be considered constant within the working range. *ω_ph_* is the frequency response of the area coverage effect of the photodiode and it depends on the working distance (or, alternatively, to the geometry of the interferogram projection), and the area of the photodiode. This causes the difference between acceleration amplitudes corresponding to $A_{s}\left(0.8801A_{v}\omega_{ph}-1\right)$, which can be seen in Figure 27.

The following observations were made from these tests:

1. The proposed methodology can be used to detect the vibrational spectrum of the microstructure with a 17.5 nm amplitude resolution in a frequency range from 5 Hz up to 13 kHz due to the shaker limitation.
2. The response of the microstructure to inertial stimuli can be adjusted by geometric modifications to improve the resolution in a smaller frequency range or to increase the frequency range but reducing the resolution.
3. An operational probe can be designed and manufactured from materials that can be used at higher than 85 °C without significant variations in response
4. It is feasible to design and manufacture a vibration measurement system for testing under relevant conditions.

## 5. Conclusions

The design, manufacture, and testing of design C, as a sensitive element for vibration detected with a Michelson interferometer, were evaluated due to the design’s better response at lower frequencies compared to designs A and B in the frequency range detection of the optic system, showing the largest displacement. Additionally, the mechanical mounting elements were manufactured, while the interferometer was assembled with commercial components. From the test results, it is concluded that:

The probe was designed to be used in an environment temperature higher than 85 °C, and its performance has been validated. In addition, according to [31], Young’s modulus variations in silicon, the material from which the microstructure is made, are insignificant at 200 °C. Therefore, the system is expected to operate at this temperature with an estimated variation in vibration amplitude of around 2%.

The optical detection system is limited in frequency, mainly by the bandwidth of the shaker. Disregarding the microsensor physical limitations, it is possible to detect signals with a maximum resolution of 17.5 nm, and up to 13 kHz. The use of a photodiode and shaker with a wider bandwidth can increase this range, increasing the potential applications. The optical scheme, using an interferometer, allows for immunity to electromagnetic interference, radio frequency interference and microstructure detection at a distance that is suitable for measurements in closed systems with a large temperature gradient. One example is for vibration in machinery that dissipates heat in specific components. Additionally, the test of the encapsulated sensor is aimed to be implemented with an optical fiber that can be used in the future to increase the distance, with low attenuation, from the vibration probe and the photodiode, similarly to what has been reported in [32].

During microfabrication, an SOI (Silicon On Insulator) substrate was not used in favor of monocrystalline silicon wafers that are etched on both sides with an in-house process. This trades additional uncertainty in the resulting microstructure thickness for a more economical and flexible process. The flexibility lies in the possibility of obtaining different spring thickness.

It can be generally concluded that, through the present proof of concept, it is possible to obtain, with microfabrication and optical detection, an efficient low-frequency vibration probe.

As future work, the accelerometer of design C can be adjusted to various dimensions of the probe mass, which can even be implemented with circular shapes; these facts would allow obtaining devices to operate with other frequencies and capabilities.

## Figures and Tables

**Table 1:** Accelerometer Dimensions.

Parameter	Description	Size (µm)		
		Design A	Design B	Design C
*H_m_*	Mass thickness	378	362	490.36
*L_m_*	Mass length	1500	1500	1500
*W_1_*	Beam width	106.9	69.55	86.02
*W_2_*	Joint width between supports and mass	83.6	94.15	96.21
*L_2_*	Length of the joint between supports and mass	86.6	124.2	86.6
*H_b_*	Beam thickness	19.4	11.4	18.9

**Table 2:** Technical Details of FEA.

Device	Solver Target	Element Type/Mesh	Convergence			
			Total Number of Nodes	Total Number of Elements	Change %	Total Mass (Kg)
Design A	Mechanical APDL	SOLID187/Refinement controlled program (Tet10)	83,842	54,922	4.02	1.9868 × 10^−6^
Design B			157,411	100,226	3.03	1.8877 × 10^−6^
Design C			19,845	8577	0	1.7624 × 10^−6^

**Table 3:** Frequencies of First Six Resonance Modes.

Vibration Mode	Frequency (Hz)		
	Design A	Design B	Design C
1	1390.6	2445.4	1007.7
2	2232.6	3672.8	2481.3
3	2235.4	3676.3	2481.6
4	49,157	53,924	7307.7
5	49,212	53,943	7308.4
6	75,224	95,190	16,444

**Table 4:** Comparison of geometrical sizes from design and the fabricated Design C.

Parameter	Description	Design C		Error, %
		Designed Sizes (µm)	Fabricated Sizes (µm)	
*H_m_*	Mass thickness	490.36	401.98	18.02
*L_m_*	Mass length	1500 × 1500	1500.13 × 1497	0.01 × 0.20
*W_1_*	Beam width	86.02	89.43	3.96
*W_2_*	Joint width between supports and mass	96.21	96.87	0.69
*L_2_*	Length of the joint between supports and mass	86.6	81.46	5.94
*H_b_*	Beam thickness	18.9	51.14	180.99

## Appendix A

Code in Octave for light exposition calculation of photodiode area.

% Data initialization lw = 8.36; % Light width in pixels dw = 2.79; % Dark widht in pixels fr = 6; % Fringe repetitions wp = 4; % Frige projection width % Varible initiallization cx = 0; cy = 0; a1 = 0; a2 = 0; a3 = 0; a4 = 0; a5 = 0; a6 = 0; % Sensor size = 2.29; % Size (square) of sensor xs = wp/2; % Sensor center x point ys = fr*(lw + dw)*0.7; % Sensor center y point theta = 0.5; % Sensor rotation angle refp = [xs ys;xs ys;xs ys;xs ys;xs ys]; sensor_nr = [ xs-size/2 ys-size/2 xs + size/2 ys-size/2 xs + size/2 ys + size/2 xs-size/2 ys + size/2 xs-size/2 ys-size/2 ]; % Sensor area rotation R = [cos(theta) − sin(theta); sin(theta) cos(theta)]; sensor = refp + (sensor_nr-refp)*R; % Step calculation samples = 1000; range = 4*(lw + dw); step = range/samples; av = zeros(samples,1); at = av; at2 = av; av2 = av; index = 1; % Base shadow sh0 = [ 0 0 ; wp 0 ; wp dw ; 0 dw ; 0 0]; for j = 0:step:range % Shadows generation sh1 = [sh0(:,1) sh0(:,2) + 0*(lw + dw) + j]; sh2 = [sh0(:,1) sh0(:,2) + 1*(lw + dw) + j]; sh3 = [sh0(:,1) sh0(:,2) + 2*(lw + dw) + j]; sh4 = [sh0(:,1) sh0(:,2) + 3*(lw + dw) + j]; sh5 = [sh0(:,1) sh0(:,2) + 4*(lw + dw) + j]; sh6 = [sh0(:,1) sh0(:,2) + 5*(lw + dw) + j]; % Intersection [rx1,ry1] = oc_polybool(sensor,sh1,’and’); i = numel(rx1); if(i > 0) a1 = polyarea(rx1(2:i-1),ry1(2:i-1)); endif [rx2,ry2] = oc_polybool(sensor,sh2,’and’); i = numel(rx2); if(i > 0) a2 = polyarea(rx2(2:i-1),ry2(2:i-1)); endif [rx3,ry3] = oc_polybool(sensor,sh3,’and’); i = numel(rx3); if(i > 0) a3 = polyarea(rx3(2:i-1),ry3(2:i-1)); endif [rx4,ry4] = oc_polybool(sensor,sh4,’and’); i = numel(rx4); if(i > 0) a4 = polyarea(rx4(2:i-1),ry4(2:i-1)); endif [rx5,ry5] = oc_polybool(sensor,sh5,’and’); i = numel(rx5); if(i > 0) a5 = polyarea(rx5(2:i-1),ry5(2:i-1)); endif [rx6,ry6] = oc_polybool(sensor,sh6,’and’); i = numel(rx6); if(i > 0) a6 = polyarea(rx6(2:i-1),ry6(2:i-1)); endif av(index) = a1 + a2 + a3 + a4 + a5 + a6; at(index) = j; a1 = 0; a2 = 0; a3 = 0; a4 = 0; a5 = 0; a6 = 0; index = index + 1; endfor
